# Anti-viral and pro-inflammatory functions of Toll-like receptors during gamma-herpesvirus infections

**DOI:** 10.1186/s12985-021-01678-x

**Published:** 2021-11-08

**Authors:** Marta Maria Gaglia

**Affiliations:** Department of Molecular Biology and Microbiology, Tufts University School of Medicine, Boston, MA 02111 USA

**Keywords:** Toll-like receptors, KSHV, HHV-8, EBV, MHV68, Gammaherpesvirus, Immune evasion

## Abstract

Toll-like receptors (TLRs) control anti-viral responses both directly in infected cells and in responding cells of the immune systems. Therefore, they are crucial for responses against the oncogenic γ-herpesviruses Epstein-Barr virus and Kaposi’s sarcoma-associated herpesvirus and the related murine virus MHV68, which directly infect immune system cells. However, since these viruses also cause lifelong persistent infections, TLRs may also be involved in modulation of inflammation during latent infection and contribute to virus-driven tumorigenesis. This review summarizes work on both of these aspects of TLR/γ-herpesvirus interactions, as well as results showing that TLR activity can drive these viruses’ re-entry into the replicative lytic cycle.

## Introduction

The oncogenic human γ-herpesviruses Epstein-Barr virus (EBV) and Kaposi’s sarcoma-associated herpesvirus (KSHV) need to overcome the immune system’s protective responses to establish lifelong latent infections in patients and to intermittently re-enter the lytic cycle. While the ability to cause a latent infection allows these viruses to persist, the lytic replicative cycle is key for spread within and between hosts. Moreover, both types of infection are important for the development of γ-herpesvirus-linked diseases [1]. EBV infection is largely ubiquitous, but if acquired past childhood EBV often gives rise to infectious mononucleosis [2]. In a subset of infected individuals, EBV infection leads to the development of various types of B cell lymphomas, as well as specific subtypes of nasopharyngeal carcinoma (NPC) and gastric carcinoma [3]. In addition, EBV infection has also been linked to the development of autoimmune diseases [4]. KSHV infection is less prevalent, with a striking geographical bias, whereby seropositivity is much higher in Africa and some other regions of the world [5]. In some infected individuals, KSHV can give rise to Kaposi’s sarcoma and two B cell lymphoproliferative diseases, primary effusion lymphoma (PEL) and Multicentric Castleman disease (MCD), as well as KSHV-associated inflammatory cytokine syndrome (KICS) [3]. These diseases are predominantly found in immunosuppressed patients, although an endemic form of Kaposi’s sarcoma not clearly related to immunosuppression is also common in parts of Africa [5].

**Fig. 1:**
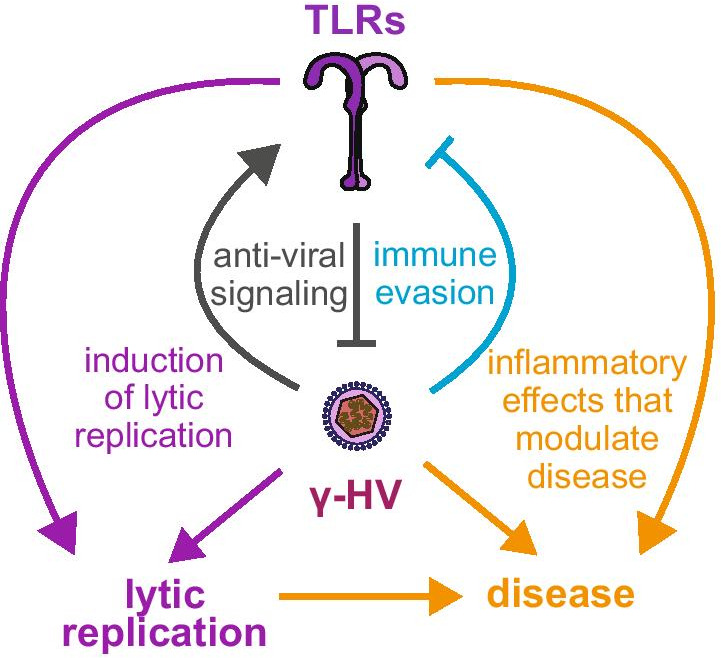
Summary of potential functions of TLR signaling in γ-herpesvirus infection. TLRs contribute to anti-viral responses against γ-herpesviruses, and their expression is induced in some situations, presumably as a result of anti-viral signaling (grey arrows). However, γ-herpesviruses also reduce expression and signaling of TLRs as an immune evasion strategy (light blue arrow). In addition, TLRs modulate γ-herpesvirus-caused diseases through their effect on inflammation (yellow arrows). Moreover, TLR activation can also reactivate the lytic cycle of γ-herpesviruses in latently infected cells (purple arrows)

Infected cells are endowed with multiple mechanisms to sense the infection and initiate a local and systemic response. In turn, viruses have evolved a plethora of approaches to counteract and limit these responses. In this review, we will focus on the interactions between γ-herpesviruses and Toll-like receptors (TLRs), a class of membrane-associated pathogen-associated molecular pattern (PAMP) receptors that detects PAMPs in the extracellular space and in endosomes. Interestingly, while many studies have studied the anti-viral functions of TLRs in γ-herpesvirus infections and viral TLR evasion strategies, interactions with TLR signaling are also relevant for other aspects of γ-herpesvirus biology (Fig. 1). Several studies suggest that TLR activation may be one of the triggers for lytic reactivation. Moreover, TLR modulation of inflammatory responses has been linked to tumorigenesis and autoimmunity, particularly during EBV infection. This review will summarize the current knowledge on these aspects of TLR biology during infection with the human viruses EBV and KSHV. I will also cover studies on the related murine herpesvirus 68 (MHV68). As EBV and KSHV do not infect mice, MHV68 is widely used as a small animal model virus for these infections, and is used to dissect γ-herpesvirus biology in vivo [6].

## Basics of TLR signaling

**Fig. 2:**
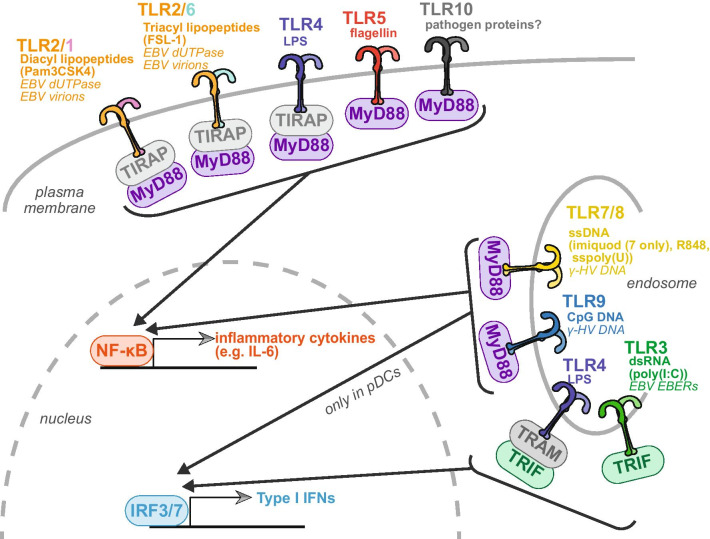
Summary of human TLRs and their ligands. Diagram of the 10 human TLRs, indicating their adaptor molecules and the downstream result of their signaling. For each TLR, canonical ligands are listed in bold, artificial stimuli used to test their functions in parentheses, and described ligands from γ-herpesviruses (“γ-HV”) in italics. pDC = plasmacytoid dendritic cells; poly(I:C) = polyinosinic:polycytidylic acid; LPS = lipopolysaccharide. Sspoly(U) = single-stranded polyuridylic acid

Mammalian TLRs are a family of transmembrane pathogen-recognition receptors (10 in humans and 12 in mice) discovered in the mid-1990s (Fig. 2). They have leucine-rich repeat containing ectodomains and cytosolic Toll-IL-1 receptor (TIR) domains. They are named for their homology to the Toll protein in *Drosophila melanogaster*, which has roles in development. Different TLRs respond to different PAMPs (Fig. 2). However, they all trigger signaling pathways that culminate in the activation of the transcription factors NF-κB and/or interferon regulatory factor 3 and 7 (IRF3 and IRF7, Fig. 2). NF-κB induces transcription of pro-inflammatory cytokines like interleukin-6 (IL-6), while IRF3 and 7 induce transcription of type I interferons (IFNs), which are potent anti-viral cytokines. In addition to responding to different cues, different TLRs have different localization, either at the plasma membrane or internal membranes, predominantly endoplasmic reticulum, endosomes and endolysosomes. Many of the internal TLRs respond to nucleic acids, which are not exclusively pathogen molecules. Their localization is thought to naturally limit the autoactivation of these TLRs by endogenous DNAs and RNAs [7]. Despite many differences in signaling, all TLRs use one of two adaptor proteins to transduce their signaling – MyD88 or TRIF (Fig. 2). Both proteins contain TIR domains. Two additional adaptors, TRAM and TIRAP, are also involved in some of the signaling, although they are thought to act mostly as sorting adaptors, helping to recruit MyD88 and TRIF to the correct locale [7].

While intracellular PAMP-recognition receptors like RIG-I and cGAS are expressed in most if not all cell types, the expression of many TLRs is more restricted and they are found predominantly on cells of the immune system [8]. Because of this distribution, they have an important role in orchestrating organism-wide responses and modulating adaptive immunity [8]. In naïve B cells, which are the critical long term reservoir of EBV, KSHV and MHV68 infections, TLR activation promotes B cell activation [9], and may be sufficient for naïve B cell proliferation without B cell receptor ligation [10]. TLR stimulation, particularly CpG DNA activation of TLR9, also leads to proliferation in memory B cells [11] and increased antibody production in plasma cells [12].

Although activation of inflammatory cytokines and IFNs appears to be the main consequence of TLR activation, at least two TLRs, TLR3 and TLR4 have been reported to directly cause cell death [13, 14]. They do so by activation of caspase-8 through formation of a TRIF-containing signaling complex [13, 14]. However, this process has largely been described in response to purified TLR ligands and it remains unclear if and when it is invoked during pathogen infection.

## TLR signaling as anti-viral mechanism and evasion during γ-herpesvirus infection

Because of the central role of TLRs in anti-viral responses, many studies have investigated which and how TLRs contribute to innate immune responses to γ-herpesviruses (Table 1). They have uncovered roles both in the infected cells themselves and in other cells of the immune system that are key to the response to infection, like monocytes and dendritic cells. TLR2, 7 and 9 (but not TLR3) are required for responses to MHV68 ex vivo in dendritic cells and in vivo [15–19]. However, TLR9 only has a role in protecting animals from MHV68 infection when MHV68 is administered from the intraperitoneal or intravenous route, not the intranasal route [16, 18, 19], possibly because TLR9 is not expressed in lung dendritic cells. Depending on the dose of the inoculum, loss of TLR9 in vivo can increase the number of latently infected cells in the spleen and/or the frequency of reactivation of lytic infection in these cells [16, 18, 19], both parameters that are important for tumor development in human herpesviruses. Consistent with the reports that TLRs sense and contribute to blocking MHV68 infection, MyD88 knockout mice were reported by Michaud et al*.* to have higher MHV68 infection rates [15]. However, a second study of *MyD88 -/-* mice reported instead that MyD88 loss impaired MHV68 infection, perhaps due to a role for MyD88 in B cell activation [17]. Possibly, these differences are linked to the dose of the inoculum, as proposed by Michaud et al*.* [15]. It is notable that inoculation doses used in the TLR studies summarized above vary by 2–3 orders of magnitude. The dynamics of the host response and/or the effect on different cell types may be different depending on the strength of the stimulus. In general, these studies highlight the complexity of the system, as TLR signaling occurs in both myeloid cells and B cells and can have different biological roles depending on the cell type.

The data available for anti-viral effects in KSHV and EBV infection are more limited. TLR9 also induces type I IFN responses in dendritic cells after KSHV and EBV infection [20–22]. It also mediates IL-17 secretion after peritoneal injection of EBV DNA in mice or ex vivo mouse monocyte treatment with EBV DNA [23]. TLR4 is activated during KSHV infection of lymphatic endothelial cells, the cells that give rise to KS, likely by the envelope glycoprotein [24]. This activation stimulates type I IFN production [24]. Moreover, loss of TLR4 increases the susceptibility of these and other cell types to KSHV infection [24]. TLR2 signaling is activated by the EBV dUTPase BLLF3, likely secreted in extracellular vesicles (exosomes), and/or a virion component [21, 25–27], and TLR3 and 7 signaling are activated by Epstein–Barr virus-encoded small RNAs (EBERs), likely also secreted by infected cells in exosomes [21, 22, 28]. Stimulation of these TLRs by EBV cues induces type I IFN and cytokine responses in monocytes and dendritic cells and promotes plasmacytoid dendritic cell maturation [21, 25–28]. The TLR mediated signaling in dendritic cells may be crucial to promote survival to infection through activation of NK and T cells, as shown by a study in a humanized mouse model of EBV [29]. Also, consistent with an important role for TLR signaling in EBV responses, a patient with a MyD88 mutation was reported to have persistent EBV viremia, albeit asymptomatic [30]. Like other data on the role of TLRs, these data point to a role for TLR signaling in protective responses against KSHV and EBV, and also highlight the range of signals from these viruses that can elicit a TLR response.

In addition to TLR activation by γ-herpesviruses, the anti-viral role of TLRs against these viruses is demonstrated by the fact that stimulation of TLRs reduces replication and reactivation of KSHV, EBV and MHV68 (Table 1). In particular, several studies have shown that TLR9 activation reduces EBV and MHV68 reactivation from a latent infection in B cells and EBV infection and/or replication in B cells and monocytes after a de novo infection [19, 21, 31–33]. The EBV studies did not test other TLR ligands, whereas Haas et al*.* also found an effect of TLR7 (but not TLR3) ligands on MHV68 reactivation in B cells [19, 31–33]. Seemingly in contrast with these results, Doyle et al*.* reported that TLR3 and TLR4 activation reduces MHV68 replication after de novo infection in bone marrow macrophages, whereas TLR9 activation in these cells has no effect [34]. However, the reported differences are most likely due to the expression levels of different TLRs and/or the downstream signaling that they elicit in the different cell types, as in macrophages TLR3/4 but not TLR9 stimulate type I IFN signaling [34]. In the case of KSHV, there may also be differences in the activity and role of TLRs depending on cell type. TLR4 activation reduces replication in endothelial cells infected de novo [24], while activation of TLR3 or TLR5 reduces spontaneous reactivation in PEL cells [35]. In addition to cytokine-based anti-viral responses, stimulation of TLR3 and 9 may also elicit another time of protective response, cell death, in latently EBV-infected cells, including some Burkitt’s lymphoma cell lines and NPC cells [36, 37]. In turn, EBV may counteract this pathway by inducing high levels of cellular inhibitor of apoptosis 2 (cIAP2) in some NPC cells, which protects them from TLR3-induced apoptosis [38]. While all these studies show a protective effect of TLR signaling, some studies suggest that TLR activation can actually *increase* γ-herpesvirus reactivation, which will be more extensively discussed in a later section.

If signaling from TLRs blocks γ-herpesvirus infection, it stands to reason that infection itself may modulate expression of these receptors. There are reports of both increase [15, 21, 39–41] and reduction of TLR expression and/or signaling [24, 39, 42–46] after infection with KSHV, EBV and MHV68 (Table 1). The direction of the detected changes depends on the virus, the cell type and the TLR studied. There are some discrepancies that are again likely to be linked to the normal function of the TLR and its ability to exert anti-viral activity in the specific cell type. Only a couple of the studies looked at more than one TLR in the same cells [39, 42, 45], so it is difficult to compare across studies. Moreover, multiple transformed B cells lines are used for KSHV and EBV studies, further complicating the picture. One consistent observation is the downregulation of TLR9 by EBV in B cells [39, 44, 45] and the reduction of signaling from multiple TLRs during MHV68 infection of macrophages and conventional dendritic cells [42, 43]. The effect of EBV on TLR9 is also consistent with the fact that activation of this TLR reduces EBV replication [21, 31–33]. Reports of reduction in TLR expression and/or signaling by γ-herpesviruses are also generally consistent with the poor responses of many cells to these viruses [42, 43], and suggest that these viruses have evolved evasion mechanisms to deal with TLR activation. Indeed, several viral proteins have been implicated in inhibition of TLR signaling: for KSHV, the master lytic regulator ORF50/RTA [42, 47–49], the virus interferon regulatory factor-like (vIRF) proteins K9/vIRF1 [50], K11/vIRF2 [50], K10.5/vIRF3 [42], the viral macrophage inflammatory protein III K4.2 [42], the viral thymidine kinase ORF21 [42], the regulators of viral gene expression ORF31 [42] and ORF57/MTA [51] and the KSHV microRNAs, particularly miR-K5 and miR-K9 [52]; for EBV, the non-coding EBER RNAs [53], the latent protein LMP1 [44], the deubiquitinase BPLF1 [54], and the host shutoff protein BGLF5 [45, 55]. These proteins and RNAs act through a variety of mechanisms. Some may alter the levels and localization of TLRs [21, 42, 45, 55] or of downstream signaling molecules like MyD88 and interleukin 1 receptor-associated kinase 1 (IRAK1) [47–49, 52]. Others may inhibit signal transduction downstream of TLR, for example through deubiquitination of signaling proteins [54]. Of note, some of the genes in the list above have homologs across the γ-herpesviruses (the regulators of gene expression ORF50/RTA/BRLF1, ORF57/MTA/BMLF1/SM, and ORF31/BDLF4, the viral thymidine kinase ORF21/vTK/BXLF1, the host shutoff protein ORF37/SOX/BGLF5, and the deubiquitinase ORF64/BPLF1). While these genes share other functions across the viruses, they were only identified in one of the viruses as TLR inhibitors. This could represent a different virus-specific function, or may simply be a limitation of the current studies. For example, to my knowledge no systematic screen of EBV or MHV68 proteins for TLR inhibition has yet been published, precluding a direct comparison with the KSHV results [42]. Lastly, in addition to these active mechanisms of TLR inhibition, Pezda et al*.* suggested that the paucity of CpG sequences in the MHV68 genome may also be responsible for reducing the TLR9 responsivity, as it reduces the viral DNA sequences that TLR9 can recognize [43]. Nonetheless, this and other studies have also reported TLR9-dependent responses to MHV68, particularly in plasmacytoid dendritic cells [16, 18, 43], potentially because there is less viral replication in these cells and thus less expression of TLR inhibiting factors [43].

Interestingly, a recent study reports an even more complicated interaction between MHV68 and TLR signaling, whereby MHV68 takes advantage of TLR3 signaling to evade other immune responses. Shen et al*.* found that in MHV68-infected bone marrow-derived macrophages, TLR3 responses result in increased SOCS1 expression, which protects MHV68 from the anti-viral effects of IFN-γ treatment [56]. Along similar lines, TLR activation in EBV infected monocytes induces programmed death-ligand 1 (PD-L1) expression [57]. As PD-L1 reduces susceptibility to killing by cytotoxic T cells, it may protect the EBV-infected monocytes [57].

While collectively the reviewed studies point to a role for TLRs in the host protective response to γ-herpesviruses, the exact effects reported differ among the viruses and in some cases in studies using the same viruses. Since the pattern of TLR expression changes depending on the cell type and in some cases the signals elicited by TLR activation are also different, many of the apparent discrepancies are likely due to differences in the experimental setup. Discrepancies in MHV68 mouse studies are more puzzling but may be at least in part due to the choice of route of infection and inoculum size. Despite the remaining questions, it is clear that multiple TLRs protect humans and mice against γ-herpesviruses, acting both in the infected cells and of the innate immune cells that respond to the infection. Further studies, particularly systematic comparisons of the same TLR in different cell types and different TLRs in the same cell type, are needed to fully dissect the contributions of TLRs in protective responses to γ-herpesviruses.

## TLR signaling as a trigger for lytic reactivation of γ-herpesviruses

In infected individuals or model organisms, EBV, KSHV and MHV68 undergo recurrent reactivation to the lytic cycle. This process has been linked to tumor development [1, 58, 59]. The cues that stimulate reactivation are only partially known, but they are generally thought to encompass many signals that trigger the death of the infected cells. The viruses are thought to have evolved to detect conditions that will lead to the death of the latently infected cells, and to respond by re-entering the lytic cycle, so that newly formed virions can infect new cells and the virus can continue to propagate. Interestingly, several studies have suggested that TLR activation can constitute a signal that triggers lytic replication in KSHV, EBV and MHV68 (Table 1). Indeed bacterial products were shown several years ago to induce lytic reactivation of KSHV in cell culture and MHV68 in an ex vivo setting, likely by stimulating TLRs [60, 61]. A recent study suggests that production of reactive oxygen species downstream of TLR2 may mediate this effect in the oral cavity following *Staphyloccocus aureus* infections [62]. Gargano et al*.* also showed that well-characterized ligands for TLR3, 4, 5 and 9 can induce MHV68 reactivation from latently infected B cells in culture, in explanted splenocytes, and even in vivo [63]. This effect did not extend to other TLRs despite similar downstream signaling. Lytic infection is stimulated after TLR stimulation because the treatment induces activation and proliferation of the B cells [63]. This effect is similar to that of B cell receptor ligation, which also induces MHV68 and EBV reactivation [61, 64]. Interestingly, in mice TLR-induced reactivation leads to an increase in the population of latently MHV68-infected cells [63]. The authors attributed this increase to increased viral production and seeding of new infections, and speculated that this may be a way in which the latent reservoir is maintained in the presence of heterologous infections [63]. However, a later study suggested that the MHV68 latent reservoir could be expanded after TLR9 stimulation also in the absence of active replication [65]. In KSHV-infected PEL cells, stimulation of the TLR7/8 with single-stranded polyU and other artificial ligands or vesicular stomatitis virus infection induces lytic reactivation [35]. Although multiple other TLRs are expressed in PEL cells, this study did not detect significant increases in lytic reactivation after activation of other TLRs [35]. Nonetheless, another group found that TLR3 activation directly increases RTA expression in a transfection setting [66], suggesting other TLRs may induce KSHV lytic reactivation in other conditions. In the case of EBV, virus reactivation and EBV-linked hepatitis were reported in a patient with infection with the syphilis pathogen *Treponema pallidum,* a known inducer of TLR2 [67]. In addition, stimulation of multiple TLRs can induce expression of the lytic regulator ZEBRA in EBV latently infected cell lines [68]. Collectively, these studies underscore the multiplicity of functions that TLR signaling can have during γ-herpesvirus infection, including a role in promoting lytic replication. As TLR signaling can act as an activating stimulus for B cell, this positive effect on reactivation may be particularly pronounced in B cells, where reactivation is also coupled to other activating stimuli.

## TLR signaling and inflammation in connection to cancer and other diseases caused by EBV and KSHV

Although much of the research on TLR and γ-herpesviruses has focused on their antiviral roles, another aspect of TLR signaling is the regulation of inflammation. Inflammation has been connected to disease progression and cancer development in both EBV and KSHV infections. Moreover, TLRs regulate B cell responses, proliferation and antibody secretion [9–12], which means that TLR stimulation could promote viral persistence and expansion of infected cells. Indeed, TLR9 inducing cues have been used to improve immortalization of EBV infected B lymphocytes in cell culture [69]. Moreover, in experimental de novo EBV infections treatment with TLR ligands increased EBV-driven B cell activation and proliferation [70, 71]. Some reports also suggest that EBV-transformed B cells express more TLR9 and TLR10 [72], although other reports found the opposite [39]. The EBV oncogenes LMP1 and LMP2A can also increase expression and responses of subset of TLRs, which provides a survival advantage to B cells and NPC cells, although these studies were done using overexpression and not EBV infection [73, 74]. In the context of NPC, the EBERs may induce TLR3 expression and cytokine-driven responses to promote tumorigenesis, as TLR3 knockdown reduces formation of NPC tumors in nude mice [75]. However, the relationship between TLR signaling and tumorigenesis may be more complex than these studies suggest. For example, a study of Finnish patients with NPC found that the low levels of TLR7 and 9 were correlated with worse disease outcomes, while lower levels of TLR5 were correlated with better outcomes [76].

Data on TLR and tumorigenesis in KSHV is limited. In a model of KSHV transformation using rodent mesenchymal stem cells, TLR4 activity was linked to increased proliferation, cytokine secretion and tumorigenesis in mouse xenografts [77]. Moreover, these KSHV-immortalized cells had increased TLR4 and MyD88 levels, suggesting the virus actively promotes their induction in the process of tumorigenesis [77]. Another potential connection between TLR signaling and KSHV tumors was identified in PEL cells, where Yang et al*.* reported that interleukin 1 receptor-associated kinase 1 (IRAK1), which acts downstream of TLRs, promotes PEL cell survival and is commonly mutated in PEL samples [78]. Moreover, MyD88/IRAK signaling is constitutively active in PEL cells [79].

EBV infection may also contribute to the development of autoimmune diseases [4]. The effect of EBV infection in autoimmunity has been linked to increased IFN responses downstream of TLR activation in some studies of scleroderma [46, 80], myasthenia gravis [81] and lupus [82]. In particular, TLR7 activation in EBV-transformed B cells may potentiate both LMP1 expression and responses to agonists of other TLRs, amplifying inflammation and potentially contributing to pathology in lupus patients [82].

Many of the studies on cancer and autoimmune disease modulation by TLR in the context of γ-herpesviruses show that these effects are mediated by increased TLR levels or signaling. This may seem paradoxical, since increased TLR activation would be expected to help clear infections. A potential explanation for these observations is that while activation of TLR early in γ-herpesvirus infection of a new host or during lytic reactivation has an antiviral function (see earlier section), activation of this pathway once latency has been established may promote disease progression. This could occur as a result of inflammatory cytokine expression, or because reactivation is blocked, favoring persistence of the latently infected cells.

## Genetic associations between TLR polymorphisms, γ-herpesvirus infection and γ-herpesvirus-linked diseases

Several studies have identified polymorphisms in TLRs that modify susceptibility to immune and infectious diseases [83, 84]. In the context of γ-herpesviruses, there have been positive and negative findings on the connection between TLR polymorphisms and susceptibility to EBV infection and disease. In general, single-nucleotide polymorphisms that reduce TLR activity or expression were found to increase infection and/or disease risk in these studies. Polymorphisms in the TLR9 coding region or promoter have been linked to metrics of EBV infection and disease in various patient groups. These include risk of EBV infection, levels of EBV in the blood, development of infectious mononucleosis, development of oral cancers, NPC tumor size and survival of NPC patients [85–88]. Polymorphisms in the TLR4 coding region and 3’ UTR have also been reported to modify risk of infectious mononucleosis, NPC and oral cancers [85, 88, 89]. In addition, in one study polymorphisms in TLR2 affected the levels of EBV DNA in the blood of infectious mononucleosis patients and the development of specific symptoms [85]. In contrast, a study of Northern Chinese gastric carcinoma patients found no connection between known polymorphisms in TLR2, 3 and 9 and susceptibility to EBV-associated gastric carcinoma [90].

Less in known about the connection of TLR polymorphisms and KSHV infection and diseases. Two studies investigated the TLR4 Asp299Gly polymorphism and found it is more common in HIV-positive patients with MCD than ones with KS or cancers unrelated to KSHV [24], and it is also more frequent in patients of African descent [91]. MCD development may be linked to poorer control of KSHV replication, as these patients also had higher viremia [24, 91]. This is consistent with the fact that the Asp299Gly sequence change results in lower level of TLR4 at the plasma membrane and lower LPS-triggered IFN induction [24, 91]. However, a recent genome-wide association study for both EBV and KSHV infection in an African cohort did not identify any TLR genes among their hits [92].

## Conclusions

EBV, KSHV and MHV68 all interact with TLRs during the course of infection, particularly as they infect cells of the immune system that express multiple TLRs. Multiple components of these viruses appear to be recognized by different TLRs, including both proteins and nucleic acids (Fig. 2). As illustrated in this review, there is extensive evidence that TLRs can act in anti-viral fashion against γ-herpesviruses. However, TLR activation can also control the latent-lytic switch in B cells, acting to promote viral replication. Moreover, TLR activity may be connected to tumorigenesis and development of autoimmune diseases in response to γ-herpesvirus infection, because of the key role of TLRs in modulating inflammatory responses. These findings suggest that modulation of TLR signaling could be used as a potential therapeutic intervention for EBV or KSHV, perhaps combined with other treatments. However, leveraging the role of TLR will require a much more precise and comprehensive understanding of the role of specific key TLRs in the dynamics of infection and disease. Currently, there are inconsistencies in the literature that need to be resolved within the study of each of the three viruses reviewed here. Many of these inconsistencies may stem from complex biology of TLRs and the multiple functions these receptors have in different cell types. However, this remains to be thoroughly tested by comparing the effects of different TLRs in different cell types in a systematic fashion. In addition, there are contradicting results in the in vivo infection results with MHV68. The in vivo MHV68 infection system is currently the best small animal model for γ-herpesviruses, and the only one available to examine the effect of TLRs at different stages of infection in a whole organism. A more systematic comparison of TLR activation and function at different inoculum doses would begin to address potential confounds in this system. Additional studies like these may point to the potential usefulness of TLR agonists and antagonists for anti-viral and/or anti-tumor therapies.

**Table 1:** Summary of studies on TLR activity during γ-herpesvirus infection

TLR	Localization and stimulus	Activated + protective role during infection?	Artificial stimulation blocks viral replication?	Activity or expression inhibited by infection?	Artificial stimulation triggers reactivation?
1/2	Cell surface; Diacyl lipopeptides (Pam3CSK4)	Activated – EBV [21, 26]	No – KSHV[35]	Yes – EBV [44, 45]	Yes – KSHV [62]
		Yes – MHV68 in vivo [15]	No – EBV [31]	No – EBV [39]	Yes – EBV [67, 68]
			Minor effect – MHV68 in cells [34]	Yes – MHV68 in cells [42]	
2/6	Cell surface; Triacyl lipopeptides(FSL-1)	Activated – EBV [21, 26]	No – KSHV[35]	Yes – EBV [44, 45]	Yes – KSHV [62]
		Yes – MHV68 in vivo [15]	Minor effect – MHV68 in cells [34]	No – EBV [39]	Yes – EBV [67, 68]
				Yes – MHV68 in cells [42]	
				No – higher—MHV68 in vivo [15]	
3	Endosome; dsRNA (polyI:C)	No – MHV68 in vivo [17]	Yes – KSHV[35]	Dynamic changes – KSHV [41, 50]	No – KSHV[35]
		Activated but aids viral replication – MHV68 in cells [56]	Yes – MHV68 in cells [34]	Yes – EBV [44]	Maybe – EBV [68]
4	Cell surface and endosome; LPS, lipidA	Yes – KSHV [24]	No – KSHV[35]	Yes – KSHV [24]	Yes – MHV68 ex vivo [63]
		No – EBV [26]	No – EBV [31]	No – higher – KSHV [77]	No – KSHV[35]
			Yes – MHV68 [34]	Yes – MHV68 in cells [43]	Yes (bacterial products) – KSHV [60]
					Maybe – EBV [68]
					Yes – MHV68 in cells, ex vivo, in vivo [63]
5	Cell surface; flagellin		Yes – KSHV[35]	Yes – EBV [44, 76]	No – KSHV[35]
					Yes – MHV68 in cells, ex vivo [63]
7	Endosome; ssRNA (sspolyU, imiquimod, R848)	Minor effect – EBV [21]	No – KSHV[35]	No – higher – KSHV [41]	Yes – KSHV[35]
		Minor effect – MHV68 in cells and in vivo [16]	No – EBV [31]	Yes — EBV [44, 45]	
			Yes – MHV68 in cells [19]	No – higher – EBV [39]	
			Maybe (promotes latency) MHV68 in vivo [19]	Yes – MHV68 in cells [42]	
8	Endosome; ssRNA (sspolyU, R848)	Activated – EBV [80]	No – KSHV[35]	No – higher – KSHV [41]	Yes – KSHV[35]
			No – EBV [31]	No – higher – EBV [80]	
				Yes – MHV68 in cells [42]	
9	Endosome; dsDNA (CpG)	Activated – EBV [22, 45]	No – KSHV[35]	No – higher – KSHV [41]	No – KSHV[35]
		Yes – MHV68 in cells and in vivo [16, 18, 19]	Yes – EBV [31–33]	Yes—EBV [39, 44, 45]	Yes – MHV68 in cells, ex vivo, in vivo [63, 65]
			No – MHV68 in cells [34]	No – higher– EBV [21, 31, 72]	
			Yes – MHV68 in cells [19]	Yes – MHV68 in cells [42, 43]	
10	Cell surface; Pathogen proteins?			Yes—EBV [39, 45]	
				No – higher in transformed cells – EBV [72]	

Only studies using infectious viruses (not single proteins) are included. TLR2 can be found in complex with either TLR1 or TLR6. Hence, studies on TLR2 are cited for both complexes, as the responsible complex is not usually clear

## Data Availability

Not applicable.

## Abbreviations

cGAS: Cyclic GMP-AMP synthetase
cIAP2: Cellular inhibitor of apoptosis 2
EBERs: Epstein–Barr virus-encoded small RNAs
EBV: Epstein Barr virus
IFN: Interferon
IL-6: Interleukin 6
IRAK1: Interleukin 1 receptor-associated kinase 1
IRF: Interferon regulatory factor
KICS: Kaposi’s sarcoma-associated herpesvirus-associated inflammatory cytokine syndrome
KS: Kaposi’s sarcoma
KSHV: Kaposi’s sarcoma-associated herpesvirus
LMP: Latent membrane protein
LPS: Lipopolysaccharide
MCD: Multicentric Castleman Disease
MHV68: Murine herpesvirus 68
MTA: mRNA transcript accumulation
MyD88: Myeloid differentiation primary response 88
NPC: Nasopharyngeal carcinoma
NF-κB: Nuclear factor κ-light-chain-enhancer of activated B cells
pDC: Plasmacytoid dendritic cells
PD-L1: Programmed death-ligand 1
PAMP: Pathogen-associated molecular pattern
PEL: Primary effusion lymphoma
poly(I:C): polyinosinic:polycytidylic acid
RIG-I: Retinoic acid-inducible gene I
RTA: Replication and transcription activator
Sspoly(U): Single-stranded polyuridylic acid
TIR domain: Toll/interleukin 1 receptor domain
TIRAP: Toll/Interleukin-1 receptor domain-containing adapter protein
TLR: Toll-like receptor
TRAM: TRIF-related adaptor molecule
TRIF: TIR-domain-containing adapter protein inducing interferon-β
vIRF: Viral interferon regulatory factor
